# Close to the metal: Towards a material political economy of the epistemology of computation

**DOI:** 10.1177/03063127231185095

**Published:** 2023-07-10

**Authors:** Ludovico Rella

**Affiliations:** Durham University, Durham, UK

**Keywords:** materiality, artificial intelligence, blockchain, cryptocurrency, hardware, GPU, ASIC, TPU

## Abstract

This paper investigates the role of the materiality of computation in two domains: blockchain technologies and artificial intelligence (AI). Although historically designed as parallel computing accelerators for image rendering and videogames, graphics processing units (GPUs) have been instrumental in the explosion of both cryptoasset mining and machine learning models. The political economy associated with video games and Bitcoin and Ethereum mining provided a staggering growth in performance and energy efficiency and this, in turn, fostered a change in the epistemological understanding of AI: from rules-based or symbolic AI towards the matrix multiplications underpinning connectionism, machine learning and neural nets. Combining a material political economy of markets with a material epistemology of science, the article shows that there is no clear-cut division between software and hardware, between instructions and tools, and between frameworks of thought and the material and economic conditions of possibility of thought itself. As the microchip shortage and the growing geopolitical relevance of the hardware and semiconductor supply chain come to the fore, the paper invites social scientists to engage more closely with the materialities and hardware architectures of ‘virtual’ algorithms and software.

## Introduction: A metonymic turn on digital materialities

Hardware limitations influence research through action: Computer scientists like to think that they think in abstract and hope that the hardware will one day support their idea, but their thinking is always limited by the hardware we have at our disposal. (LeCun, 2019, minute 11-12:30)

Yann LeCun, Silver Professor of the Courant Institute of Mathematical Sciences at New York University, Vice President, Chief AI Scientist at Meta, and pioneer of deep learning, in this speech at the International Solid-State Circuits Conference of 2019, lays bare an often-unexplored link not only between software and hardware, but between abstraction and material implementation that traverses debates on computation. The idea that computer scientists ‘think through hardware’ (see Munn, 2022a), as the quote seems to suggest, was not lost on Alan Turing, for whom thinking and calculating is always performed through machines and hardware. Here representing the computer as a person, rather than as a machine, he writes: ‘every … operation consists of some change in the physical system consisting of the computer and his tape’ (Turing, 1937, p. 250). Indeed, ever since the Pascaline invention by Blaise Pascal, the history of computation is also the history of tools for computation (Jones, 2016): ‘Counting or writing’ argues Bernhard Siegert, ‘always presuppose technical objects capable of performing … these operations’ (Siegert, 2015, p. 11, in Hayles, 2020, p. 32). Hence ‘by virtue of their material properties, technological artifacts are part of the normative order rather than external to it’ (Miller, 2021, p. 61).

This active role of hardware architecture has only recently started to surface in the social sciences. When it appears, hardware is mostly shown in the process of becoming waste (Gabrys, 2011; Thylstrup, 2019) or as a source of energy consumption for its production or its functioning (Taffel, 2023), and not for the specific functions that was designed to perform. While some articles have acknowledged the role that computing architectures have played as ‘material developments [that] brought forth or actualized latent algorithmic capacities’ (Grosman & Reigeluth, 2019, p. 7), only a few scholars have incorporated hardware and architectures as analytical dimensions in their own rights. Azar et al. (2021, p. 9–10) develop a sixfold stack of algorithmic vision made of a social level, a computational level, a data level, an algorithmic level, a physical level and an axiomatic level. A. Mackenzie and Munster (2019) showed how ‘*platform seeing* transpires as a new mode of invisual perception’ out of ‘the conjunction of image ensembles and artificial intelligence architectures, devices and hardware’ (p. 6, original emphasis). Gaboury’s (2021) book *Image Objects* contains an archaeology of the GPU, focusing on the graphic processing applications of this piece of hardware. Here I also look at the GPU, but explore other uses of it.

This article is based on close readings of computer science papers on hardware architectures, artificial intelligence, and cryptoassets, understood as invaluable sources of epistemological and ethico-political meaning making (Amoore et al., 2023). ‘Close to the metal’ is a term in computing science to denote a property of programming languages to directly access and influence the behaviour of hardware. In D. Mackenzie’s (2021) investigation of High Frequency Trading, C++ is often adopted because it allows programmers to ‘build a level of abstraction and then, when you need to, … just blow right through it and get down to the hardware’ (p. 167). For my concerns here, ‘close to the metal’ is also a coding environment designed to access and program graphics processing units (GPUs) for general purpose calculation (GPGPU). It was launched by the company ATI in 2006 to rival Nvidia’s CUDA, which later became the *de facto* standard for general purpose GPU computing (Lezar, 2011, p. 9).

Analytically, the need to stay ‘close to the metal’ is part of a broader need for a ‘metonymic turn’ in the study of computation. For Straube (2016, p. 6), the metonymy—the identifying a concept with a thing embodying or closely resembling that concept—operates not so much by analogy, but by ‘[taking] a real technical model (actually informing system-building practices by computer engineers), and slightly [widening] its scope while staying close to its original context’. Putting instruments like GPUs at the front and centre of analysis means acknowledging the specificity of their functioning and their impact they have in meaning- and money-making. Staying close to the metal means to investigate the affordances and the limits imposed by the ‘*materialities* of information’, that is, ‘those properties of representations and formats that constrain, enable, limit and shape the ways in which those representations can be created, transmitted, stored, manipulated, and put to use’ (Dourish, 2017, p. 26 original emphasis). In line with this need for specificity, Amoore (2020, p. 24) has recently expressed wariness with ‘‘algorithm talk’ when it is asserted generally and without specificity, for different algorithms are as varied in their logics and grammars as languages are, and these differences … should be made to matter’. The same wariness animates my concerns in this article, only from the point of view of hardware.

Graphic Processing Units (GPUs) are ‘knowledge machines’ (Galison, 1997, p. 63), and ‘epistemic infrastructures’ (Munn, 2022a, p. 1399) that enable specific computational practices while also foreclosing other ones, or making some other practices necessary as a result. On the one hand, mass-scale parallelization affords GPUs to act as ‘multipliers’ (Easterling, 2014) over multiple computational domains, such as cryptoasset mining and artificial intelligence. This multiplying capacity derived from a pre-existing political economy of computer gaming which propelled the development of retail high-performance parallel computing for graphic processing. In turn, the uptake in GPUs for crypto mining further fostered increased efficiencies and competition over architecture designed which made GPUs even faster. When it comes to machine learning, the increased performance of GPUs allowed them to expand dramatically the use cases in this industry, also due to the ‘data hungry’ nature of both GPUs and machine learning algorithms. At the same time, however, GPUs would not have been able to play the role that they did if artificial intelligence and machine learning had not changed their epistemological foundations, with a shift away from symbolic AI and towards connectionism. In short, GPUs show how materiality, political economy, and epistemology can never be fully separated from each other, but combine in producing ‘cognitive assemblages’ that transcend industries and computational domains.

## Machines, power and thought

Each new machine that is built is an experiment. Actually, constructing the machine poses a question to nature; and we listen for the answer by observing the machine in operation. Newell and Simon (1976, p. 114)

Hardware in the social sciences is evoked to show how ‘the cloud’ has its own topologies and topographies (Hu, 2015) and environmental impacts (Atkins et al., 2021; Lally et al., 2022) determined by cables (Starosielski, 2015), datacentres (Pickren, 2018), often co-located with older infrastructures, such as telegraph or pneumatic mail pipes (Blum, 2012). When individual devices are analysed, they are treated as artifacts that have specific cultural and social lives, rather than internal logics and material agency, besides the study of the waste and pollution that goes *into* turning minerals into hardware (Crawford, 2022), or that are generated when hardware itself *becomes* waste (Gabrys, 2011). Materiality then, is often used to ‘ground’ digitality, or to show how in both material and immaterial, analogue and digital environment, big data industry retains the same extractive logic: extraction of minerals, extraction of data. Literature in infrastructure studies, media studies and digital geography has indeed talked about the ‘global assemblage of digital flow’, but the unit of analysis there is more frequently the datacentre than the individual piece of hardware (Kinsley, 2014; Munn, 2022a; Pickren, 2017).

Yet, as this paper will show, the role of hardware is not just that of being an obdurate substratum of abstract software and thought. If it is true that hardware performs a ‘mediation between a cosmic order and an inframolecular order’ (Simondon, 1992, p. 318 in Gabrys, 2016), hardware mediates by organizing thoughts and planning actions and reactions in machines. Hardware is always already epistemological, and at the same time both performance and architecture derive from political economic consideration about use cases, market valuation, and competition. As A. Mackenzie and Munster (2019, p. 5) would have it, ‘hardware [;] devices; forms of parallel computation; and computational architectures … constitute (nonhuman) activities of perception’. Hence, ‘the becoming environmental of computation’ (Gabrys, 2016) is not only about sensors, but about sense-making devices and cognitive assemblages, if by cognition we understand the ‘process of interpreting information in contexts that connect it with meaning’ (Hayles, 2020, p. 6).

This transformation lies at the basis not only of the growing planetary assemblage of sensing equipment, but also of the growing relevance of data analytics hardware on the same devices that gather images—for example, smartphones that are simultaneously optimized for image quality *and* machine learning processing (A. MacKenzie & Munster, 2019, p. 15). The materiality of hardware is ‘Einsteinian’ (D. MacKenzie, 2021, p. 11), in that the ‘materiality of the small’—the microchip—is in no way subordinate to the ‘materiality of the large’—computers, datacentres, companies, markets. At the micro level, the speed of light, or electrons, in a medium, and the heat generated by the friction in that medium, are key determinants of the ‘floorplan’ of a microchip in terms of transistor density, cooling, and memory access speed, in turn generating macro-effects in terms of energy consumption and need for cooling equipment. Just as routing and packet size standards generated specific business structures and topologies in the Internet (Blanchette, 2011; Dourish, 2017), GPUs and parallel computing are here shown to have emerged primarily as a result of the external capitalization forces of the videogame industry and, subsequently, cryptoasset mining.

This materiality has effects also on what forms of thought are enabled and disabled, prioritized and discarded (Kornberger et al., 2019; Munn, 2022a). This epistemological relevance of hardware architectures is important not only to adjudicate whether, and in which ways, specific types of material support can allow the emergence of cognition and intelligence (Fazi, 2019), but also to show how the logic of those architectures channel, change and co-opt human cognition and intelligence in specific ways (Mühlhoff, 2020). Navigating this tension between epistemology and political economy has required, over time, hybrid ‘philosophical entrepreneurs’ who ‘sought to make the most advanced natural philosophical and artisanal knowledge of the day pay off in practical applications for state and markets alike’ (Jones, 2016, p. 98). Importantly, as it will be shown in the ‘mangle’ of epistemology and political economy (Pickering, 1995), the role of the philosopher and that of entrepreneur always coexist in this field, and forms of thoughts and regimes of valuation combine in determining which technologies emerge. Indeed, as Galison (2003) shows, the standardization of time and space measurement was an epistemic *and* politico-economic effort spearheaded, simultaneously, by astronomical observatories, on one side, and telegraph and railway industries, on the other. Indeed, this paper draws on the invaluable contribution coming from literature on the history of hardware that has shown that material logic is but one component of technological development: competition logic and market logic—and I would add geopolitical logic—play just as important roles (Brock & Lécuyer, 2012; Lécuyer & Brock, 2010). Semiconductors and their political economy are now receiving attention from economics and economic geography scholars (Prytkova & Vannuccini, 2022; Yeung, 2022), but no research to date has combined this structural analysis with a ‘close to the metal’ view into the inner logic of individual devices.

D. Mackenzie (2021) summarizes his approach as material political economy: material, in that more-than-human materialities have a degree of political agency over the structures they impact on, and they spatialize these relationships in specific ways. Political, because that agency always already constitutes forms of constrains to other agent’s actions, possibilities, and propensities. Economy, because the material political assemblages are leveraged at extracting resources and profits or at altering the distribution of resources and profits generated elsewhere. To this conceptualization, this paper adds epistemology: ways of knowing are influenced by material, political and economic influences, and vice versa. What this paper is seeking, echoing Galison, is not a material political eco-epistemology *of* machines, but *in* machines (Galison, 1997, p. 26). As the next three sections will show, market dynamics connected with videogames and cryptocurrencies pushed GPUs from expensive and unprogrammable hardware to cheap, powerful, and malleable parallel computers. In turn, this newly afforded computational power opened up new ways of seeing and knowing the world, at the basis of present-day turns to machine learning. If the planet-scale network of datacentres represents a macro-scale knowledge and epistemic infrastructure (Edwards et al., 2013; Munn, 2022a), hardware devices such as the graphic cards explored here bring that analysis at a micro level, by showing how ‘investments in forms’ (Kornberger et al., 2019, p. 1) give shape to knowledge (Mattern, 2020, in Munn, 2022a) even at a nanometre scale.

## Graphic processing units between videogames and parallel computing

When a long series of identical computations is to be performed, such as those required for the formation of numerical tables, the machine can be brought into play so as to give several results at the same time. (Menabrea, 1843, pp. 689–690)

The passage quoted above is by Italian polymath and politician Luigi Menabrea and translated by Ada Byron Lovelace, commenting on Charles Babbage’s Analytical Engine in 1843, and it shows that parallel computing is as old as computation itself. Parallel computing can be summarized as ‘a collection of processing elements that cooperate and communicate to solve large problems fast’ (Culler et al., 1998, p. 20), and it can take two main forms: instructions parallelism and data parallelism. Instruction parallelism allows executing instructions in parallel. Data parallelism consists in performing different or the same instructions on individual elements in a larger data structure, such as individual values in arrays and matrices (Lezar, 2011, p. 7). Following Flynn’s (1972) taxonomy of instruction architectures, while CPUs are either Single Instruction, Single Data if single-core, or Multiple Instructions, Multiple Data if multi-core, GPUs are Single Instruction, Multiple Data. In short, a parallel computer takes as input not one single number or piece of data, but an array—vector—or a table—matrix, or a higher dimensional tensor, and then performs on them a set of instruction to output not just one but multiple numbers simultaneously.

**Figure 1.**
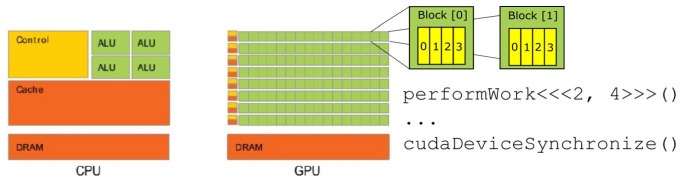
Comparison of CPU and GPU architectures. *Source*. Thambawita et al. (2014, p. 1).

Graphics processing units (GPUs) are integrated circuits specialized in the production and rendering of images, dating back to 1970s consoles and workstations. Central processing units (CPUs) are effective at performing a large number of operations *in sequence* on the same data, while GPUs are extremely effective at computing the same calculations on a large number of datapoints *simultaneously*. Figure 1 provides a diagrammatic comparison between a CPU and GPU Each green square in the GPU’s diagram represents a Streaming Multiprocessor (SM). The instruction performWork<<<x, y>>>() assigns computing resources to a given function (Kirk & Hwu, 2013). After having run the function, cudaDeviceSynchronize() exports the results calculated by the GPU to the CPU.

GPUs are organized in this way because of the needs of image processing, particularly in video and other dynamic and three-dimensional settings. An image is decomposed into a matrix of pixels—for example, a sphere represented in 3D graphic. To render the smoothness of that sphere the different pixels are mapped onto ‘primitives’, that is, different triangles, and they are differently shaded so that the eye does not perceive triangles as such, but different light gradients in the lighting of the texture mapped (Owens et al., 2008, pp. 880–881). Parallelism enables seeming real time dynamism because it makes possible to change the value of all pixels simultaneously.

**Figure 2.**
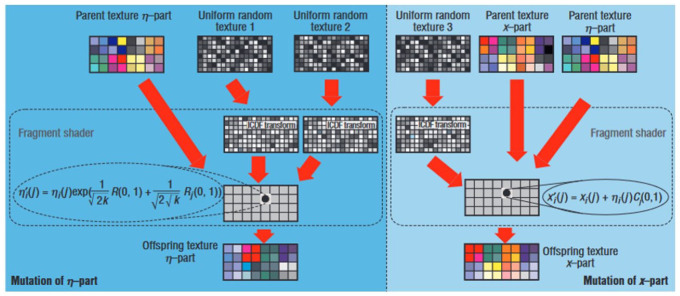
First use of GPU on non-visual data. *Source*. Fok et al. (2007, p. 73).

While the term ‘graphics processing unit’ dates back to 1969 and the LDS-1 system by Evans & Sutherland in 1969 (Gaboury, 2021, p. 168), the acronym GPU only emerged in 1999 with the NVidia GeForce 256 (NVIDIA, 1999). Originally GPUs were ‘configurable but not programmable’ (Dufrechou, 2021, p. 4; Owens et al., 2008, p. 2): software engineers had to convert mathematical operations into graphic shading operations, and transform tables and matrices into textures (Figure 2). As demand for General Purpose GPU (GPGPU) for scientific research grew, large GPU manufacturers started opening up their system to non-graphic computation: Between 2006 and 2008, Nvidia and ATI launched their own standard programming frameworks for GPGPU, respectively the Compute Unified Device Architecture (CUDA) and Close to Metal (CTM; AMD, 2006; Kirk & Hwu, 2013, p. 35). Subsequently, AMD abandoned CTM for the open standard OpenCL (AMD, 2010).

While the ‘Einsteinian materiality’ of GPUs (D. Mackenzie, 2021) exerts agency on the development of this technology, it is important not to take at face value overly deterministic ideas around the origins and trajectories of silicon developments. In fact, trade-offs between, for example, transistor density, energy consumption and computing power are often framed as natural laws: ‘Moore’s law’, ‘Blinn’s law’ and ‘Dennard’s law’ are considered natural limits to, respectively, transistor density, power density and computing time. Trade-offs and limitations set by the materialities of the small, often connect to the very hard limit set by the speed of light in any given medium, serve as ‘multipliers’ or ‘switches’ (Easterling, 2014, p. 71), enabling and disabling macro-phenomena. Moore’s law, which states that computing power will double every year, was extrapolated from specific trends in specific industries and gradually adopted by the semiconductor industry through the authoritativeness of Moore himself, but also through the market power of Fairchild and Intel (Lécuyer, 2022). In D. Mackenzie’s (2006) words, we could say that Moore’s law was an engine, not a camera: It created dynamics that it purportedly only explained. This is not to say that Moore’s law has no value: As far as the imperative for semiconductors is make general-purpose parallel computing hardware ever denser, this density generates problems in terms of overheating and faults. The materiality of the device, then, exerts a power of ‘disposition’, that is ‘a propensity within a context’ as Easterling (2014, p. 71) would say.

The arms race around the micro-materialities of GPUs to generate denser and higher-performance chips produced a highly oligopolistic market. Hence, since the 1990s, Nvidia acquired 3dfx, maker of the then-leading Voodoo chip (Kanellos, 2002) and AMD acquired ATI, maker of the Radeon GPU line (CNW Group, 2007). Intel tried to stay relevant by integrating its own graphic chips onto motherboards, obligatory pieces of hardware that in turn host CPUs, hard drives and other components. At present, 16 firms produce GPUs worldwide contending a market worth $78.56 billion in 2020 and $86.78 billion in 2021, of which $13 billion come from workstation GPUs and $36 billion from the gaming computer segment of the market, up from, respectively, $7 and $18 billion in 2012 (Aslop, 2021a, 2021b; The Business Research Company, 2021). As of 2022, out of the Top 500 list of supercomputers, NVIDIA provides graphic acceleration for a combined 92.46% of the total 146 devices that use accelerators (TOP500, 2020). Nvidia and AMD, with market capitalizations of, respectively, $430.21 billion and $153.42 billion, are virtually tied between 18% and 20% of this market, with Intel—market cap $176.15 billion—making up 60%—especially through the pre-installation of Intel boards on computers that carry Intel CPUs—and around 2% is left to the remaining 13 companies (Aslop, 2021c; CompaniesMarketCap.com, n.d.). Again, this market logic is by no means determined unproblematically from the material logic of the silicon: The progressive adoption of CUDA as the *de facto* standard in terms of GPGPU had significant import in locking in Nvidia’s dominance over this industry.

The same micro-materiality that produced those macro political economies produced another trend, that is, the move away from general-purpose hardware accelerators, like GPUs, to Application-Specific Integrated Circuits (ASICs). In fact, ever denser parallelism produces problems connected with the cooling of the chip that are called ‘dark silicon’ (Taylor, 2013a), that is, the need to switch off portions of the microchip to avoid overheating and loss of efficiency (Pias et al., 2019, p. 14). As computation becomes more complex, computational gains derive less and less from sheer parallelism and more from the optimization of the surface of the chip, etching on its surface the parts of the algorithm that are harder to parallelize, that is, ‘bespoke silicon’ (Taylor, 2013b, p. 1). This move away from GPUs is somewhat ironic, since GPUs themselves emerged as application-specific chips for graphic processing, because CPUs would not have been able to process all the pixel colours in an image at a speed that would have allowed any form of user immersion and realistic and seamless movement (Gaboury, 2021, pp. 162–163).

Hence, GPUs illustrate that there is no clear-cut division between software and hardware, between instructions and tools, but also between thoughts and the conditions of possibility of thought itself. Hardware and software are in dialectic unity and unitary tension whereby

[i]t is impossible to ‘add’ software to hardware, or data to code—they each exist on separate conceptual planes and are, in themselves, lacking nothing … Each layer depends on the one below to function, and adds a dimension of abstraction that is in turn the base for the layer above. (Straube, 2016, p. 6)

This layered understanding of computation is echoed, in computer science and hardware-software architectures, by ‘hardware-software co-design’:

The process of learning computer architecture is frequently likened to peeling an onion … At each level of understanding we find a *complete whole* with many interacting facets, including the structure of the machine, the abstractions it presents, the technology it rests upon, the software that exercises it, and the models that describe its performance. (Culler et al., 1998, p. 21 emphasis added)

## Blockchain: A material political economy of parallel computation

A cryptocurrency is a digital asset operating in a distributed, time-stamped, append-only ledger, simultaneously held by all users across a decentralized network, called the blockchain. The blockchain is updated following a set of rules, instructions, and procedures called consensus algorithm (Rella, 2020). Proof-of-work is a consensus algorithm whereby specific nodes, called miners, gather transactions broadcast through the network and encrypt all these values together. The encrypted value must fall below a specific value set as a ‘difficulty level’ and, to do so, miners need to include arbitrary strings to the block called ‘nonce.’ Since it is impossible to foresee if a hash value for a given input will fall below that difficulty level before calculating it, the only efficient strategy is to try different nonce values at random.

GPUs were instrumental in the first expansion of cryptoasset mining, especially between 2011 and 2013: GPU parallelism speeds up mining by allowing miners to load different versions of the block content, each with a different nonce associated with them, and then performing in parallel the hash calculations (Orender et al., 2020, p. 2). Initially, Bitcoin miners employed regular computers’ CPUs, or on workstations. Subsequently, with the improvement of graphic cards, the first CUDA and OpenCL miners were created around 2010s (McFarland, 2010/2021). However, while GPUs were better than CPUs at parallelizing hashing algorithms, there are significant limits to GPUs’ efficiencies. Bitcoin’s SHA256 algorithm is composed of many computations to be executed in sequence, which in itself is a task for which GPUs are not fully optimized (Hayes, 2017) even though these operations per se do not create the most painful bottlenecks of GPU designs, like memory access or floating points (Taylor, 2013b, p. 5). GPUs also encountered diminishing returns due to energy requirements for cooling, their price and the speed with which they depreciated once they became obsolete (Taylor, 2017).

**Figure 3.**
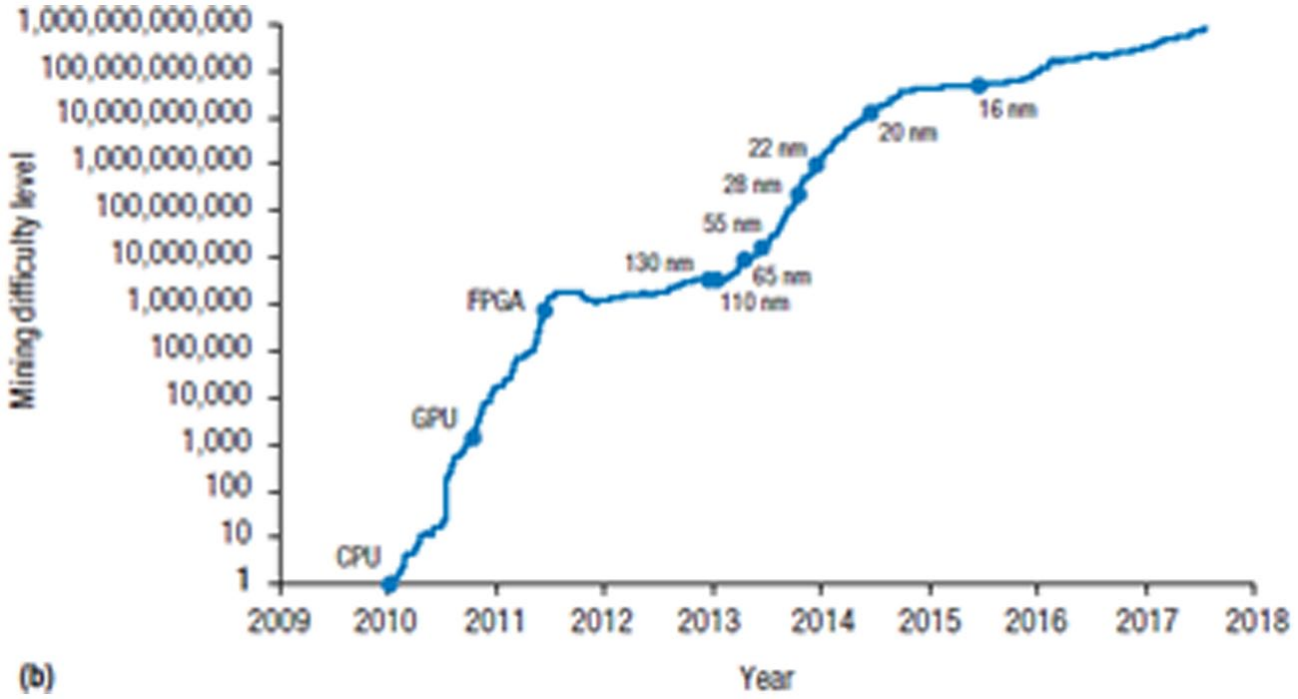
Bitcoin mining difficulty over time. *Source*. Taylor (2017, p. 60).

Hence, GPUs’ general-purpose architecture sowed the seeds of its own replacement via a combination of increased density and microchip specialization: From being the cutting-edge around 2011, crypto mining shifted from GPUs to Field Programmable Gate Arrays between 2012 and 2013 and, since 2013, on Application-Specific Integrated Circuits (ASICs; Mahony & Popovici, 2019). SHA256 is highly parallelisable, but it is also easy to encode in hardware. In turn, performance gains can be made by reducing the dimensions of the transistors responsible for carrying out the calculations, albeit with the trade-offs discussed before: ‘The only improvement for Bitcoin mining ASICs is to migrate to the latest process technologies and possibly apply custom library cells or even custom physical layout’ (Vranken, 2017, p. 5). As Fuchs and Wentzlaff (2019, p. 7) have it, ‘confined domains such as Bitcoin mining will become bound by the limited number of ways to represent the core algorithm in hardware’. Figure 3 shows the evolution of Bitcoin mining difficulty—a proxy for computational power—over time, corresponding to the introduction of different hardware architectures.

**Figure 4.**
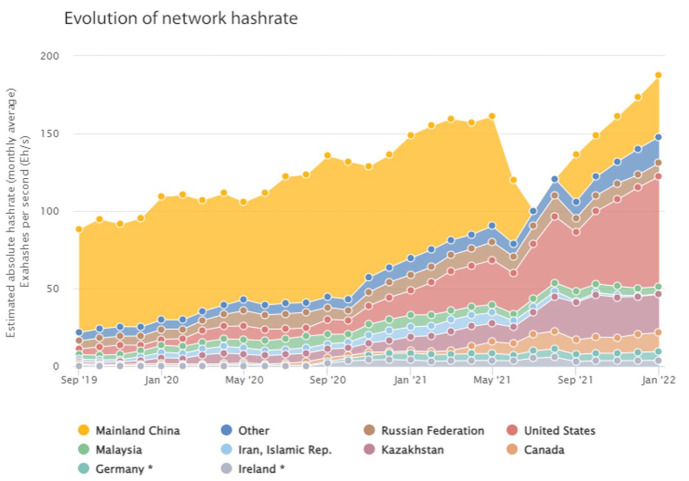
Evolution of Bitcoin hashrate since 2021. Notice the dip corresponding to the Chinese ban on mining. *Source*. Author’s own, Cambridge Centre for Alternative Finance (n.d.).

Once again, materiality produced its own political economies: Miners pooled to minimize the risk, and started accepting fees in exchange for quotas in the newly mined bitcoin (Bruschi et al., 2019). The tendency towards concentration of mining power around large ASIC clouds was met with fierce resistance in some parts of the cryptoasset community: after all, the Bitcoin white paper did envision a network run on the principle ‘One CPU, one vote’ (Nakamoto, 2019). This became painfully clear when, in May 2021, China, which always was a strict jurisdiction for crypto mining, decided to implement an outright ban on this computational practice. As Figure 4 above shows, the hash-rate decreased by one third overnight. This could theoretically reverse centralization by bringing older mining hardware back into profitable territory due to the sudden drop in the difficulty of cryptographic puzzles. However, the simultaneous take-off of hash-rate in other jurisdictions like the US and Russia indicates that mining hardware relocated rather than disappearing from the market (Tidy, 2021). Regardless of the short-term impact on the mining industry, the medium-term trend shows that the hash-rate, and hence the minimum viable hardware standards that miners need to meet, are now at the same level as they were before the ban, and there are signs that China is once again becoming an important jurisdiction for mining (Akhtar & Shukla, 2022). A full overview of the role of regulation, materiality and energy determinants in the location decision of cryptoasset mining firms is beyond the scope of this paper, but a good case study is Wyeth et al. (2023).

Due to these centralizing tendencies, as well as for environmental concerns, different cryptoassets have experimented with ASIC-resistant algorithms. One such example of partially ASIC-resistant proof-of-work algorithm was Ethereum’s consensus algorithm before this cryptoasset moved to proof-of-stake in 2022, completely abolishing mining as a means of validation. This consensus algorithm forced miners to randomly access the Ethereum blockchain to create a ‘seed’ formed by a random subset of previous block hashes, and to attach that seed to the block they intend to validate (Ward, 2020). Ethereum’s algorithm’s main bottleneck, thus, was memory access, an area of hardware development where the difference in performance between cutting-edge ASICs and comparatively cheaper cutting-edge GPUs was not as wide (Walton, 2022a). GPU mining, then, remained alive in Ethereum long after it became completely unprofitable for Bitcoin, and accounted for as much as 35% of the consumer demand for GPUs globally (Gkritsi, 2022). This in turn put competitive pressure on GPU prices across industries.

This story is not just about supply: Demand played a role in shaping GPU markets, whereby gamers and miners alike battled over the price and availability of graphic hardware. A secondary ‘scalper’ market developed, of people hoarding pricy hardware to resell in on Ebay and other retail online stores (Walton, 2022b). The relevance of mining for the GPU market put graphic card manufacturers under the spotlight of financial regulators, and it was ‘priced into’ the valuation of these firms. For example the American Securities and Exchanges Commission (SEC) fined Nvidia for $5.5 million for failing to disclose in its corporate reports the profits it derived from GPU sales destined to mining (SEC, 2022).

When Ethereum switched to proof-of-stake in September 2022, hence abolishing mining, market actors produced different outlooks for Nvidia’s future, some bleak (Pound, 2022), while others more hopeful (Saleem, 2022). Nvidia’s stock, already declining prior to the Ethereum merge, dipped in November 2022 to half the price it fetched in August 2022. However, probably because in the meantime Nvidia reinforced its position as key actor in machine learning (see below), Nvidia’s stock grew steadily since October 2022, from around $110 dollars per share to $275 at the time of writing (Google Finance, n.d.). With the end of GPU mining in Ethereum, furthermore, Nvidia decided to distance itself from this industry, arguing publicly that cryptocurrencies do not ‘bring anything useful for society’ (Hern, 2023).

## Thinking through hardware: GPUs and the making of artificial intelligence

One thing that we discovered in Bell Labs is that it is very hard to succeed, in Neural Networks, using exotic hardware … GPGPU should have come ten years earlier … people at Microsoft started experimenting on GPUs for neural nets in the mid 2000s but no one was interested in them. (LeCun, 2019, minutes 11 and 21)

Backpropagation now works amazingly well, and the reason is that now we have lots of computing power. Things like GPUs and more recently TPUs allow you to apply a lot of computation and they have made a huge difference. The deciding factor I think was the increase in compute power. Credit for Deep Learning really goes to the people who collected the big databases like Fei Li, and those who made computers go fast. (Hinton, 2019, minute 26)

The 2017 and 2018 Alan Turing Prizes were awarded to two sets of winners with remarkably complementary research interests: While 2018 winners Geoffrey Hinton, Yann LeCun, and Yoshua Bengio were focused on software, especially convolutional neural networks, 2017 winners David Patterson and John Hennessy (Hennessy & Patterson, 2019), both computer architecture scholars, focused on hardware and hardware architectures as the most important drivers for the future of computation: ‘Disjoint as it might look, this train of thoughts intersect to make a coherent whole. New advances in new deep learning algorithms and techniques capitalize on novel architectures for parallel processing’ (Pias et al., 2019, p. 9). While I have highlighted how GPUs’ materiality can enable the explosion of entire industries, a focus on AI illustrates how that material political economy inextricably links with issues of epistemology and knowledge production.

**Figure 5.**
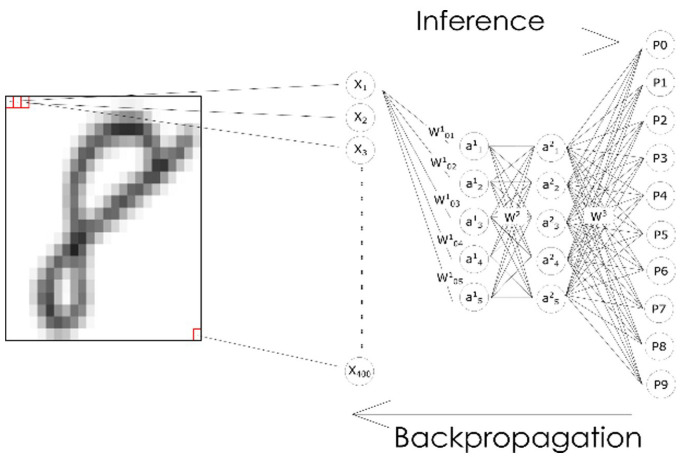
A handwritten digit parsed through a feedforward neural network. *Source*. based on LeCun et al. (n.d.) and Burrell (2016).

A neural network is a layered ensemble of mathematical functions structured as a network, with each function—called neuron—taking a set of values as input and transforming those inputs in a specific way (Figure 5). The first layer takes the vector composed of the pixel in the picture (X_1_ ~ X_400_) and multiplies simultaneously (i.e. in parallel) each and all values in it by a matrix of weights (W^1^_01-05_) and then feeding the results to a non-linear activation function in each neuron (a^1^_1_ ~ a^1^_5_). After this is performed in one layer, the results are fed as a new matrix to the matrix of weights W^2^ for the subsequent layer, and so forth until the output layers (P0-P9). The weight matrices W^1^ W^2^ and W^3^ update to minimize distance between the prediction of the algorithm and the correct prediction, based on an optimization algorithm called backpropagation (LeCun et al., 2015).

**Figure 6.**
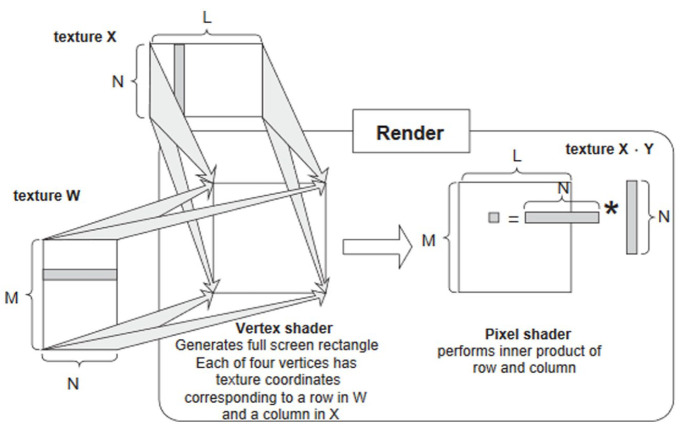
Matrix multiplication as texture rendering. *Source*. Oh and Jung (2004, p. 1313).

GPUs facilitate matrix-vector multiplication. These operations are relatively easy to parallelize by loading the vector and the matrix in the memory and then performing the vector-matrix product on different threads of the GPU’s streaming multiprocessor (Figure 6). The first implementation of neural networks on GPUs was Oh and Jung’s Feed Forward Network on an ATI RADEON 9700 PRO, reporting a 20-fold improvement in performance using rendering to perform calculation on non-visual data much like that shown in Figure 2 (Oh & Jung, 2004). Almost in the same year, Steinkraus et al. (2005) implemented a two-layer fully connected neural network on a GPU and reported a three-times speedup over their CPU-based baseline. Bengio et al. (2007) ran a Deep Belief Network that took 29 minutes to update 45 million parameters over 1 million training examples, while a CPU gear took more than a day. Chellapilla et al. (2006)’s convolutional neural network for document processing was the first character recognition system, and it reported a three- to fourfold speedup compared to CPUs. AlexNet, whose victory in ImageNet competition in 2012 triggered an ‘AI spring’ after the end of the decade-long ‘second AI winter’ (see below), found out that ‘1.2 million training examples are enough to train networks which are too big to fit on one GPU’, and decided for the first time to parallelize *across* GPUs (Krizhevsky et al., 2012, p. 3).

Daston and Galison identify a general shift across sciences from images-as-representation to images-as-process: ‘Images began to function at least as much as a tweezer, hammer, or anvil of nature: a tool to make and change things’ (Daston & Galison, 2007, p. 383). Within AI, this shift towards image as tool is exemplified by the operationalization of the image as feature vector and feature matrix on which to perform calculations: The image becomes a specific instance of the vector and matrix as a diagram to computationally apprehend both visual and non-visual data (Rieder, 2020). While this expands machine and platform vision to ‘invisual’ forms of data (A. MacKenzie & Munster, 2019, p. 6), it also de-visualizes images and turns them in just another form of data. Indeed, the explosion of Transformers, at the basis of large language models and generative models such as ChatGPT was driven by a concern with making inherently sequential objects like sentences into more picture-like matrices of tokens. Before transformers, language processing employed Recurrent Neural Networks (RNN), which generated sequences of ‘hidden states’ as function of previous hidden states given an input, storing word sequences so that the system could predict subsequent words given an input. ‘This inherently sequential nature’, researchers at Google Brain and the University of Toronto argued, ‘precludes parallelization …, which becomes critical at longer sequence lengths, as memory constraints limit batching across examples’ (Vaswani et al., 2017, p. 2). Conversely, Transformers work by ‘eschewing recurrence and instead relying entirely on an attention mechanism to draw global dependencies between input and output’, transforming a sentence in a matrix of co-dependencies between word tokens. As Munn (2022a) has shown in his study of datacentres, materiality can privilege some ways of thinking and marginalize others, and this may work at macro as well as micro scales. In this case, sequentiality was abandoned in favor of parallelism, also because of hardware configuration.

Without the massively parallel computing capacities of GPUs, computations like image recognition, let alone language processing and generation, would not have been possible, due to what Edwards (2010) has called *data friction*: Means of computation, like the material heft of punch cards, create likewise material impediments to the feasibility of some forms of knowledge about the world (Dourish, 2017). However, this is not just a story of finding the right means to a pre-given end. Rather, neural networks emerged also thanks to GPUs, but in response to a wider epistemological turn in AI. Between the 1950s and the 1980s, the leading paradigm was that alternatively called symbolic AI or Good Old-Fashioned AI (GOFAI), eventually generating the Expert System paradigm. This approach was animated by efforts to encode human logics in *if-then-else* statements that machines could understand and use to achieve general intelligence. Withing this framework, software instructions do not require a significant number of computations run in parallel, but a series to sequential deductive steps to come from premises to conclusions. Indeed, in 1958, while comparing complex computers to the human brain, the computer science pioneer Von Neumann (1958/2012, p. 51) said ‘large and efficient natural automata are likely to be *highly parallel*, while large and efficient artificial automata will tend … rather to be *serial’*. Good Old-Fashioned AI also had its own hardware accelerators, LISP machines, but they did not take off both for performance reasons, and because the investment appetite in artificial intelligence cooled down (Newquist, 2020, p. 345).

A change in attitude happened after the 1980s, with the so-called ‘AI winter’ that followed the acknowledgement that expert systems did not perform as well as promised. Under the banner of connectionism, AI resurfaced not as a formal and logical symbolic processing framework, but as a statistical framework of calculation in search for plausible reconstructions and representations of patterns in data: ‘The earliest work on planning in AI took a deductive approach [whereby] designers hoped to represent all the system’s ‘world knowledge’ explicitly as axioms’ (Dennett, 1987, p. 141). Conversely, connectionism sees any given form of knowledge as a ‘shifting coalition of microfeatures’ (Clark, 1990, p. 206) whereby an AI system begins ‘with a random distribution of … weights and connections’ and then ‘learns’ by backpropagation to adjust the weights towards the correct output: ‘in connectionist theorizing, the high-level understanding will be made to revolve around a working program which has learnt how to negotiate some cognitive terrain’ (p. 213). In short, for connectionism, law-like explanations of phenomena are not acquired knowledge that should be encoded in an intelligent machine as a pre-existing baggage of axioms necessary to navigate and negotiate the world. Rather, they are epiphenomena of low-level number churning and weight-adjustments in the process of learning matrices of parameters from the ground up. As Clark (1990, p. 221) put it, ‘In the early days of Artificial Intelligence, the rallying cry was “Computers *don’t* churn numbers, they *manipulate symbols*!” … now the wheel has come full circle. The virtue of connectionist systems, it seems, is that ‘they don’t manipulate symbols, they *crunch numbers*’’ (original emphasis). It is not a coincidence, then, that in criticizing the GOFAI approach to artificial intelligence, much of Dennett’s (1987) promise hinged on the development of ‘fast parallel processors [which] will bring in their wake huge conceptual innovations which are now only dimly imaginable’ (p. 149).

Indeed, as Amoore and colleagues have shown, while connectionism was present since the 1950s, with Rosenblatt’s model of the perceptron (Rosenblatt, 1958), and while it started to gain momentum in the 1980s, it was only in the early 2000s and some of the ‘victories’ such as the ImageNet competition, thanks to GPUs, that this epistemological approach became mainstream (Amoore et al., 2023, p. 1). Convolutional neural networks for image recognition had been performing well, but they were rejected by the scientific community because, according to Hinton, the task of AI had been to define deductively the task that the machine was meant to solve, rather than programming a machine that inductively performed well at a task (Hinton, 2019, p. 24).

In parallel with shifts in epistemology and changes in hardware performance represented by faster and faster GPUs, the interplay between AI and GPUs was changed by the production and extraction of extremely large datasets. Both machine learning and GPUs are data-hungry: While GPUs are very fast at computation thanks to small but fast caches located close to the Streaming Multiprocessors, they are relatively slow in both access to the global memory and in offloading computed data onto the so-called host memory—which is the Random Access Memory (RAM) of the computer where the GPU is installed (Nageswaran et al., 2009, p. 2147; Raina et al., 2009, p. 874). In turn, Deep Learning algorithms require a large amount of data to avoid overfitting, that is, the tendency to perform well on the data used for training, but performing far less well on new and hitherto unseen data.

Data, hardware and software evolved at least partially independently from each other. Platforms did not initially incorporate Deep Learning analytics, GPUs were powerful and ‘data hungry’ regardless of Deep Learning and actual availability of data, and, as a consequence, Deep Learning models did not have at their disposal the gargantuan amount of data that they have today. In fact, seminal models like AlexNet were originally trained on academically curated datasets such as MNIST and ImageNet. MNIST is a database of 70,000 handwritten digits, each picture being normalized into a 20 × 20 pixel image in greyscale, published in 1998 by LeCun et al. (n.d.). ImageNet is a dataset published in 2009 by Fei Li at University of Illinois Urbana-Champaign, containing 3.2 million images sorted through the crowd working platform Mechanical Turk, divided into 5,247 categories according to WordNet, in turn a semantic hierarchical database of English words dating back to the 1980s by George Miller at Princeton (Gershgorn, 2017). ImageNet became the benchmark for image recognition algorithm, and, in 2012, a Convolutional Neural Network, called AlexNet and developed by Geoffrey Hinton, Ilya Sutskever, and Alex Krizhevsky from the University of Toronto, was the first one to break the wall of 25% error in assigning a picture to the correct label.

However, as machine learning algorithms gained traction, they required and, in turn, propelled a turn towards datafication, data extraction and accumulation and, increasingly data production ostensibly *ex nihilo* through generative models for synthetic data (Jacobsen, 2023; Steinhoff, 2022). Propelled by the uptake of social media and portable devices and by broader turns across industries towards data-driven business models that made data monetization an essential component of the revenue and profitability of small and large businesses alike, datafication, ‘surveillance capitalism’ (Zuboff, 2019) and ‘surveillance advertising’ (Crain, 2021) turned towards the production of data as a commodity and as capital (Sadowski, 2019), that is, both as an asset that can be bought and sold, and as a mix of raw material and productive factor for the ‘platform political economy’ (Langley & Leyshon, 2021).

Without material support that allowed mass data processing at scale, models that are now widespread, like Convolutional Neural Networks and Transformers would not have been possible. And without an epistemological framework that envisioned artificial intelligence as being an inductive process of performing vector-matrix multiplication, parallel computing would not have been a useful device. In addition, without the mass production of data that was enabled by widespread connectivity, social media and mass production of images and text, neither the hardware nor the software would have achieved their full potential. This in turn fed into a market that, as we saw above, was highly concentrated.

Market concentration is becoming increasingly sensitive at a regulatory and political level: The UK barred Nvidia from acquiring ARM, a leading microchip IP licensing company that is especially important for low-energy microchips like mobile phones’ System-on-a-Chip and other edge devices (GOV.UK, 2022). The United States issued, on October 7th 2022, new controls for advanced semiconductor technologies and microchips manufactured in China if using transistors under 14 nm (Bureau of Industry and Security, 2022). Nvidia claimed it lost around $400 million in revenue as a result of that ban, and this contributed, together with Ethereum merge to a dip in stock value, although that value has since recovered and grown (Nellis & Lee, 2022).

This interplay between epistemology, hardware and data does not unfold seamlessly and indefinitely. While GPUs can still be significantly faster than CPUs, their capacity is limited. In the same way, back-propagation can be parallelized in a limited way, because each layer’s weights can only be updated after the previous ones have done so (Goodfellow et al., 2016, pp. 432–433). This, in turn, has triggered the need for ‘bespoke silicon’ and ASICs: Google’s Tensor Processing Units (Jouppi et al., 2017) and Nvidia’s Tensor Cores (NVIDIA, 2017) integrate so-called systolic arrays that combine matrix-matrix multiplication and accumulation, that is, that work by multiplying and adding values in sequence. Other companies are not taking Nvidia dominance without a response, as Google TPU were launched as a proprietary alternative to GPUs, and Tesla launched its DOJO chip for self-driving cars, among others (Reuther et al., 2022). On the other hand, so-called neuromorphic chips are trying to imitate the ‘energy-saving’ characteristics of the brain on a silicone basis (Khan et al., 2008), and combining it with Spiking Neural Network architectures that are not organized in layers, but rather activate individual networks based on contingent patterns of connections (Khan et al., 2008). Furthermore, the energetic footprint of datacentres, and the corresponding growth in computing power in end devices such as sensors and mobile phones, is driving a decentralization of AI computing from the ‘cloud’ to the ‘edge’ (Munn, 2022b; Narayan, 2022). However, as in LeCun’s observation that opened this section, ‘exotic architectures’ still provide hurdles to performance because they require bespoke code to allow the engineers to ‘speak’ to the hardware. Hence, the jury is still out on the increased performance of current neuromorphic chip architectures (Diamond et al., 2016).

## Conclusions: Going full stack

Full Stack: The entirety of a computer system or application, comprising both the front end and the back end. *Oxford Dictionary*

Critical hermeneutics-based approach would focus on the entire social construction of ML as an end-to-end problem, addressing not just how bias and prejudice are molded within ML models but how they are molded in those who seek ML as a solution to social problems. (Roberge & Castelle, 2021, p. 19 emphasis in original)

This article has argued that social studies of digital technologies need to increase the level of specificity in our analysis, if we want to attend to the conditions of possibility of contemporary computational practices. At a time when the materiality of digital technology comes once again to the fore because of microchip scarcity (Egan, 2021) and the geopolitical significance of semiconductor value chains (Zakaria, 2022), future research will increasingly have to pay attention to the social relevance of the ‘mangle’ (Pickering, 1995) of epistemology, materiality and political economy, such as the one that this paper unpacked. Overall, through a joint analysis of blockchains and AI, GPUs have been shown to play an essential role in the political economy and epistemology of multiple industries.

First emerging in video game industries as image processing chips, GPUs have afforded large-scale parallel hardware that influenced multiple industries, including cryptoassets and machine learning. In the case of cryptoassets, this manifested itself in an influx of capital and diversion of hardware meant for videogames towards the lucrative crypto-mining industry, but it also further spurred advancements in parallel computing and, subsequently, in domain-specific architectures such as ASICs. This, in turn, contributed to an epistemological revolution, decades in the making, in data analytics and AI. From rules-based models typical of Good Old-Fashioned AI and Expert Systems, GPUs enabled the hitherto minoritarian school of connectionism, based on matrix multiplication, to become the hegemonic force in computer science for AI. While this shift would not have happened without the emergence of Big Data, that mole of data could not have been processed without GPUs, and neither the availability of data nor the availability of computing power would have had the effects it had without an epistemological turn towards connectionist AI. In turn, this central role of graphic cards is also driving a separate arms race towards domain-specific chips like TPUs, which include a specific memory for neural network parameters, so that backpropagation can be sped up, and neuromorphic chips. This will affect the political economy of both graphic cards and machine learning models. One could argue that the change in hardware, combined with the political economy that GPU hardware contributed to generate, also caused a quasi-epistemic shift in cryptocurrency, triggering Ethereum’s choice to develop ASIC-resistant consensus algorithms and, subsequently, moving away from proof-of-work entirely to embrace proof-of-stake.

As I have shown, then, there is never an unambiguous cut between the material, the political, the economic and the epistemological: If computer scientists ‘think through hardware’ then the process of production of that hardware has just as much influence on theoretical structures as those theories can exert on hardware through technological innovation. At the same time, GPUs have participated in the epistemological endeavour of computer scientists because they are ‘dense with meaning, not only laden with their direct functions but also embodying strategies of demonstration’ (Galison, 1997, p. 2). The development of new AI hardware, especially neuromorphic chips, must be watched closely, to see which new kinds of both human and machine cognition they may afford (Fazi, 2019).

This article opens up at least two avenues for future research. First, as D. Mackenzie (2021) has noticed, every material political economy, by virtue of its materiality, produces specific ‘*spatial* materialities’ (p. 12 original emphasis). In both blockchain and AI, this spatial materiality instantiated itself in clouds understood as large-scale assemblages for ‘planet-scale computing’ (Xie et al., 2018), with Bitcoin ASICs and Google TPUs being the cutting edge of those industries. However, cloud computing is more than just an assemblage of datacentres (Amoore, 2018), but is instead a highly flexible arrangement (Narayan, 2022). By a combination of cloud and edge computing, artificial intelligence models are trained in datacentres and then deployed on mobile phones, autonomous cars, and CCTVs to perform inferences in real time (Munn, 2022b). Nvidia’s failed attempt at purchasing microchip designer company ARM was seen as gaining ground into Edge AI devices, where ARM architectures were more widely used than traditional GPUs and x86 CPUs (Nast, 2021).

Second, this paper calls for future research into the multiple material, epistemological and symbolic relationships between games and AI. In fact, the type of computations that GPUs were originally designed for—namely, realistic graphic representations of sceneries and environments—already incorporate use cases of image generation, simulation, and reinforcement learning. The type of simulation that the GPU is required to handle, then, closely resembles the artificial environment where AI agents are made to interact with each other and with human agents in reinforcement learning exercises. Materially, games produced the computing needs that fostered the arms race in computer parallelism that brought to the emergence of powerful GPUs. Epistemologically, Mirowski (2002) has noticed how games played an important role in cybernetic understandings of the economy and of its governance. GPUs have also played a role in parallelizing macroeconomic analysis (Aldrich et al., 2011; Duarte et al., 2020), and they ushered in a machine learning approach to economic and econometrics that is increasingly gaining traction in the discipline (Athey, 2019). Hence, while the victories of AI agents in video games like Go or Starcraft have attracted mass media attention (Deepmind, 2019), future research might look into, on the one hand, what residue of the logic of the game survives in the rationality underpinning AI models and, on the other hand, how games and game-like settings like digital twins become sites where new ways of understanding and governing the social are elaborated and enacted.
